# Strategies for the prevention of bronchopulmonary dysplasia

**DOI:** 10.3389/fped.2024.1439265

**Published:** 2024-07-24

**Authors:** Gianluca Dini, Sara Ceccarelli, Federica Celi

**Affiliations:** Neonatal Intensive Care Unit, Santa Maria Hospital, Terni, Italy

**Keywords:** bronchopulmonary dysplasia, neonatal care, postnatal corticosteroids, preterm infants, lung disease

## Abstract

Bronchopulmonary dysplasia (BPD) is a common morbidity affecting preterm infants and is associated with substantial long-term disabilities. The pathogenesis of BPD is multifactorial, and the clinical phenotype is variable. Extensive research has improved the current understanding of the factors contributing to BPD pathogenesis. However, effectively preventing and managing BPD remains a challenge. This review aims to provide an overview of the current evidence regarding the prevention of BPD in preterm infants, offering practical insights for clinicians.

## Introduction

Bronchopulmonary dysplasia (BPD) is the result of a complex process in which several prenatal and/or postnatal factors interfere with lower respiratory tract development, leading to a severe, lifelong disease (1). BPD was first described by Northway et al. (2) based on radiographic and histological evidence of pulmonary disease.

BPD used to be a significant concern for all preterm infants requiring prolonged invasive mechanical ventilation. However, with the widespread adoption of antenatal corticosteroids, surfactant therapy, and gentle ventilation strategies, BPD has become rare among preterm infants with birth weights greater than 1,500 g (3). Despite these advancements, recent data suggest that BPD rates have not improved and may even be increasing among extremely preterm infants (4). In this review, we summarize the available evidence for the different pharmacological agents currently in use to prevent BPD, primarily focusing on findings from randomized controlled trials (RCTs). Table 1 provides a summary of strategies commonly used for the prevention of BPD.

**Table 1:** Summary of interventions for the prevention of BPD.

Intervention	Comments
Oxygen saturation targets	Maintain oxygen saturation 90%–95%
Ventilatory strategy	Avoid endotracheal tube ventilation, encourage non-invasive support strategies (NIPPV, SNIPPV, nCPAP). Lung protective strategies: Consider volume-targeted ventilation—TV 4–6 ml/kg
Surfactant	Exogenous surfactant therapy given within the first 30–60 min after birth is effective in the prevention and treatment of RDS and reduces the need for mechanical ventilation and oxygen supplementation (risk factors for BPD). INSURE/LISA should be used for surfactant administration in spontaneously breathing infants.
Antenatal corticosteroids • IM Dexamethasone (6 mg q12 h × 4 doses) • IM Betamethasone (12 mg q24 h × 2 doses)	No evidence regarding improved BPD outcomes but improves survival. A single course of corticosteroids is recommended for pregnant women between 24 0/7 weeks and 33 6/7 weeks of gestation who are at risk of preterm delivery within 7 days, including for those with ruptured membranes and multiple gestations.
Postnatal corticosteroids ○ Early (<8 days): ▪ Hydrocortisone: (IV 0.5 mg/kg/dose q12 h × 7 days followed by q24 h × 3 days) ○ Late (>7 days): ▪ Dexamethasone: (IV/PO: 0.075 mg/kg q12 h × 3 days, 0.05 mg/kg q12 h × 3 days, 0.025 mg/kg q12 h × 2 days, and 0.01 mg/kg q12 h × 2 days)	We suggest using systemic glucocorticoids selectively in EPT infants (GA < 28 weeks) who remain ventilator-dependent at a postnatal age of two to four weeks and in whom attempts to wean from the ventilator have failed and/or who require oxygen supplementation >50 percent. In this setting, we administer low-dose dexamethasone according to the protocol used in DART (Dexamethasone: A Randomized Trial). Parents/caregivers should be informed of the risks and benefits and should participate in decision-making.
Caffeine citrate (loading dose: 10–20 mg/kg maintenance dose: 5 mg/kg/day)	Early caffeine administration (as soon as possible after birth) is recommended for all infants ≤30 weeks gestation, to be continued until PMA 34–36 weeks.
Vitamin A (IM 5,000 IU/dose 3 times per week for 4 weeks)	May be used for prophylaxis in centers with a high baseline incidence of BPD.
Nutrition	Breast milk is the preferred source of nutrition for preterm infants as it offers several advantages over formula, including the prevention of BPD.
Macrolide antibiotics • Azithromycin • Clarithromycin • Erythromycin	May be used in research setting only.

BDP, bronchopulmonary dysplasia; EPT, extremely preterm; GA, gestational age; IM, intramuscular; INSURE, intubation-surfactant-extubation; IV, intravenous; LISA, less invasive surfactant administration; nCPAP, nasal continuous positive airway pressure; NIPPV, Nasal intermittent positive pressure ventilation; PMA, postmenstrual age; PO, orally; TV, tidal volume.

### Definitions of BPD across the ages

**Figure 1:**
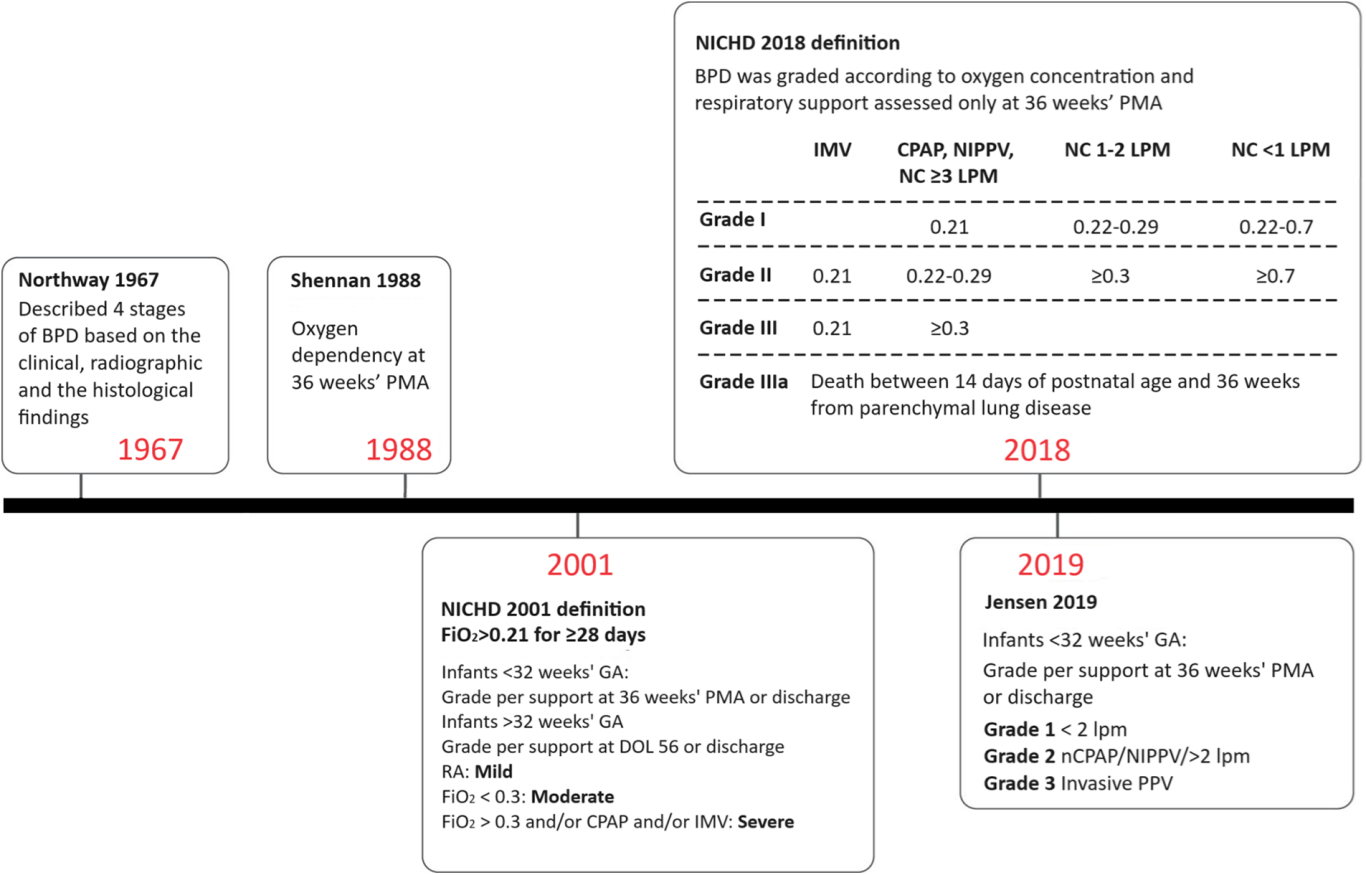
Definitions of BPD over time. BPD, bronchopulmonary dysplasia; CPAP, continuous positive airway pressure; DOL, day of life; FiO_2_, fraction of inspired oxygen; GA, gestational age; IMV, invasive mechanical ventilation; LPM, liters per minute; NC, nasal cannula; NIPPV, nasal intermittent positive pressure ventilation; PMA, postmenstrual age; RA, room air.

BPD was first described in moderately preterm infants during a time when supplemental oxygen was the primary treatment for severe respiratory distress syndrome (RDS) and mortality rates exceeded 50%. Since then, the definition of BPD has evolved (Figure 1), largely due to the improved survival rates of extremely preterm infants and the development of new support modalities.

The original definition of BPD was published by Northway in 1967. Affected infants were born during the late saccular or alveolar stages of lung development and were exposed to high oxygen concentrations and high peak airway pressures during mechanical ventilation (2, 5). Northway described four stages of BPD based on clinical, radiographic, and histopathological findings (6). Preterm infants with BPD initially presented with acute respiratory distress syndrome, which evolved into a chronic disease after day 28 of life. Chest radiograph findings correlated with histopathological changes, showing an initial acute inflammatory stage followed by chronic inflammation resulting in fibrosis.

The 2001 National Heart, Lung, and Blood Institute (NHLBI) workshop definition took the important step of including a BPD severity scale (7), and infants with severe BPD were subsequently found to have higher mortality and rates of adverse outcomes after discharge than those with mild or moderate disease (8). However, the definition of severe BPD was overly broad, and combined infants receiving 31% oxygen by low flow nasal cannula with infants on high ventilator settings and receiving drugs for pulmonary hypertension. This issue was partly addressed by the NHLBI 2018 revision, which reserved grade 3 BPD for infants receiving positive pressure or nasal cannula flow >3 L/min in addition to oxygen (9).

Most recently, Jensen et al. analyzed 18 prespecified BPD definitions based on the respiratory support level and supplemental oxygen at 36 weeks postmenstrual age (PMA) to determine which definition best correlated with early childhood morbidity. The study demonstrated that a grading system based on respiratory support, regardless of oxygen level, best predicted late death or serious respiratory morbidity at 18–26 months corrected age (10). The Jensen definition classifies BPD severity in infants at 36 weeks PMA into grade 1 for those requiring 2 L/min nasal cannula or less, grade 2 for those requiring more than 2 L/min nasal cannula or other forms of non-invasive ventilation support, and grade 3 for those requiring invasive mechanical ventilation (10).

Several limitations remain with the revised definition (11). Firstly, the decision for respiratory support still lies with the clinician. These decisions are often based on subjective assessments of the infant's work of breathing, frequency of apnea/bradycardic events, ability to take adequate oral feeds or maintenance of an optimal growth trajectory. At 36 weeks’ PMA, preterm infants may still exhibit immature respiratory control, manifesting as periodic breathing or apneic events. The use of diuretics (12) and bronchodilators (13), which can influence the need for respiratory support, varies markedly between centers (14) and may affect the reporting of BPD. Additionally, the revised definition does not differentiate infants with BPD-associated pulmonary hypertension (BPD-PH).

### Epidemiology

#### Prevalence

Premature birth (<37 weeks gestational age) is common and affects 6%–14% of pregnancies, depending on the country (15, 16). Premature infants are generally classified based on gestational age [extremely preterm infants or extremely low gestational age newborns (ELGANs) are <28 weeks gestation; very preterm infants are 28 to <32 weeks gestation, moderate preterm infants are 32 to <34 weeks and late preterm infants are 34 to <37 weeks] or birth weight (ELBW infants are <1,000 g, very-low-birth-weight (VLBW) infants are <1,500 g, and low-birth-weight (LBW) infants are <2,500 g). BPD incidence increases as gestational age and weight at birth decreases. BPD remains the most common complication associated with prematurity and is increasing in prevalence, most likely due to the increased survival of ELGANs (17, 18). Data from major cohort studies (such as ELGAN, Canadian Neonatal Network, Korean Neonatal Network, Vermont–Oxford Network, and Swiss Neonatal Network, as well as studies in China, Taiwan, and India) demonstrate a BPD prevalence of 11%–50%, a wide range that is due to differences in gestational age or birth weight criteria for a BPD diagnosis (19–25).

#### Risk factors

The strongest risk factors for BPD are prematurity and low birth weight (26–29). Nearly 80% of infants born at 22–24 weeks of gestation are diagnosed with BPD (30), while only 20% of those born at 28 weeks develop the condition. Among infants with BPD, 95% are VLBW (31). Other perinatal risk factors include intrauterine growth restriction (IUGR) (21), male sex (21, 29), and, inconsistently, chorioamnionitis (32), race or ethnicity (21, 27, 29), and maternal smoking (33, 34). Genetic factors may also contribute to BPD development, as suggested by twin studies (35, 36), and research is ongoing to identify specific genetic markers associated with BPD (37, 38).

### Evaluation

The evaluation of BPD involves assessing blood gases, chest x-rays, and the nutritional status of the patient (39). An arterial blood gas analysis may reveal hypoxia, hypercarbia, or acidosis. Patients with BPD are monitored with continuous pulse oximetry to maintain adequate oxygen saturation levels. Many centers also use transcutaneous carbon dioxide monitoring to assess the infant's ventilation.

**Figure 2:**
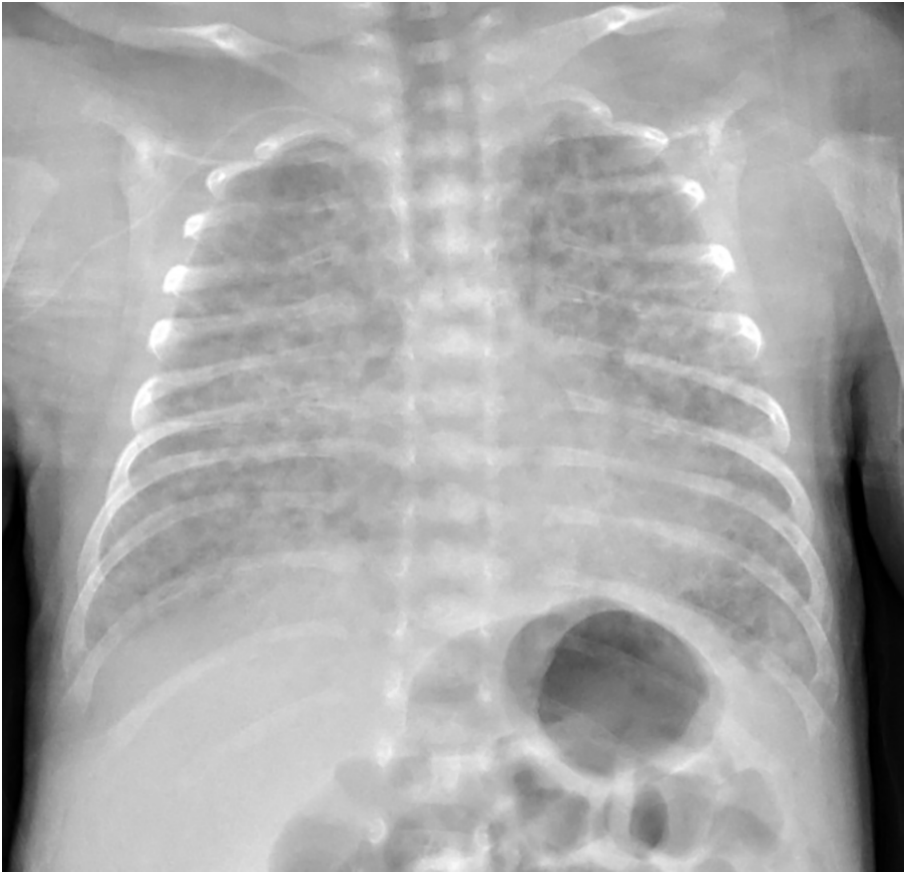
Chest radiograph of an infant with bronchopulmonary dysplasia.

Among imaging modalities, plain chest radiography and computed tomography scanning remain the most extensively studied in BPD (40). Chest radiographic features of established BPD include interstitial thickening, focal or generalized hyperexpansion, and atelectasis (Figure 2) (41). High-resolution CT scans can reveal abnormalities not easily seen with routine chest radiography. Although computed tomography findings were more specific for BPD than those of plain radiographs (42, 43), the considerable exposure to radiation, increased cost of the procedure, and need for either patient cooperation or deep sedation have limited the use of computed tomography. The European Respiratory Society (ERS) guidelines recommend follow-up imaging with ionizing radiation only in the most severely affected patients (44).

In contrast, ultrasound does not require exposure to ionizing radiation and can be performed at the patient's bedside. An ultrasound scoring system involving the evaluation of three different areas of the lung with a semi-quantitative score has shown some potential for predicting moderate to severe BPD (45).

Infants with moderate or severe BPD should be screened for pulmonary hypertension (PH) at 36 weeks PMA using an echocardiogram (46, 47). Some centers choose to screen all patients with BPD for PH due to the high morbidity and mortality associated with this condition. There are known limitations in the sensitivity and specificity of echocardiography to identify PH, and cardiac catheterization is recommended when there are uncertain findings, concerns for inadequate response to pulmonary vasodilators, or more precise measures of pulmonary pressures and vasoreactivity are needed (48).

### Lung protective ventilation strategies

Successful transition to postnatal breathing requires the clearance of fetal lung fluid and adequate lung aeration. However, several factors in very preterm infants can hinder this process, including high chest wall compliance, weak respiratory muscles, incomplete surfactant production, and underexpression of transepithelial sodium channels (49–51). As a result, many very preterm infants require positive airway pressure and supplemental oxygen shortly after birth to maintain physiological stability. While invasive mechanical ventilation can be life-saving in these cases, it also poses a risk of lung injury. Animal studies have demonstrated a clear connection between baro- and volutrauma induced by mechanical ventilation and pathological changes in the lung resembling BPD (52, 53). Additionally, observational studies have suggested a link between invasive mechanical ventilation and an increased risk of BPD (26, 54). To mitigate lung injury and prevent BPD, researchers have investigated various noninvasive and gentler invasive ventilation strategies. Salient data from randomized controlled trials on the effects of various respiratory support strategies for prevention are summarized in Table 2.

**Table 2:** Summary of randomized, controlled trial data on the effects of various respiratory support strategies for preventing death and/or bronchopulmonary dysplasia.

Intervention	Outcome	Trials/N	Outcome rates		Relative risk (95% CI)
			Intervention	Control	
Respiratory support strategies					
nCPAP vs. MV (55)	Death or BPD	4/2,782	40%	43%	0.90 (0.83–0.98)
sNIPPV vs. nCPAP after extubation (56)	BPD	3/181	28%	43%	0.64 (0.44–0.95)
Volume-targeted vs. pressure limited MV (57)	BPD among survivors	9/620	23%	35%	0.68 (0.53–0.87)
HFOV vs. pressure-limited MV (58)	BPD among survivors	17/2,786	30%	35%	0.86 (0.78–0.96)
	Death or BPD	17/3,329	41%	45%	0.90 (0.84–0.97)
Surfactant administration					
LISA vs. INSURE (59)	BPD	13/1,758	10%	17%	0.65 (0.51–0.82)

BPD, bronchopulmonary dysplasia; HFOV, high-frequency oscillatory ventilation; INSURE, intubation-surfactant-extubation; LISA, less invasive surfactant administration; MV, mechanical ventilation; N, total number of infants evaluated for the outcome; nCPAP, nasal continuous positive airway pressure; sNIPPV, synchronized nasal intermittent positive pressure ventilation.

#### Noninvasive positive airway pressure

One strategy to prevent ventilator-induced lung injury is to avoid mechanical ventilation altogether. Three large RCTs have compared early noninvasive continuous positive airway pressure (CPAP) with immediate intubation and surfactant administration. Despite variations in design elements, including the gestational ages of enrolled infants and initial CPAP settings (ranging from 5 to 8 cm H_2_O), each study demonstrated a nonsignificant reduction in the rate of death or BPD at 36 weeks’ PMA among infants initially treated with CPAP (60–62). Meta-analyses of the available trial data, some of which also included smaller RCTs, demonstrated a small but statistically significant reduction in the risk for death or BPD with CPAP therapy (Table 2). Although one large trial reported higher rates of pneumothorax in CPAP-treated infants, meta-analyses did not show an increased risk for pneumothorax or other adverse events with early CPAP (55, 63, 64). Consequently, the American Academy of Pediatrics Committee on Fetus and Newborn recommends early use of CPAP with subsequent selective surfactant administration in extremely preterm infants as an evidence-based strategy to reduce the risk of death or BPD (65).

Heated and humidified high-flow nasal cannula (HFNC), typically administered with flow rates higher than 1 to 2 L/min, has gained popularity as an alternative to nCPAP. HFNC offers potential advantages such as reduced nasal trauma, simpler device setup, and greater facilitation of oral feeding and skin-to-skin care (66). However, recent trial data suggest that HFNC and nasal CPAP may not be equivalent therapies. Treatment failure is more common among very preterm infants who receive HFNC compared with nasal CPAP as a primary support modality. While HFNC may be an acceptable alternative to nasal CPAP for post-extubation support among infants born at 28 weeks of gestation or more, routine use in less mature infants is not recommended (67).

Nasal intermittent positive pressure ventilation (NIPPV) is a form of non-invasive ventilation that delivers a baseline distending pressure similar to CPAP but with the addition of superimposed peak inspiratory pressures at intervals (68). NIPPV may be synchronized (SNIPPV) or non-synchronized to the infant's breathing efforts. A Cochrane analysis of ten studies including 1,061 infants comparing early NIPPV and early CPAP use determined that even though infants randomized to early NIPPV had reduced risk of requiring intubation (RR 0.78 CI 0.64–0.94) and respiratory failure (RR 0.65 CI 0.51–0.82), there was no reduction in the risk of BPD among infants who received NIPPV (69). Another meta-analysis of the use of NIPPV vs. CPAP in preterm infants after extubation found a reduction in BPD associated with synchronized NIPPV (RR 0.64, 95% CI 0.44–0.95) on subgroup analysis, but in the overall cohort no difference was found in the rates of BPD between the two groups (RR 0.94, 95% CI 0.80–1.10) (56).

#### Mechanical ventilation

Mechanical ventilation remains an essential tool in the care of critically sick and very preterm infants, despite improvements in perinatal care including increased use of antenatal steroids and non-invasive respiratory support. Studies from CPAP trials suggest that as many as 65% of spontaneously breathing extremely preterm infants may still require intubation and mechanical ventilation despite early CPAP therapy (55). In such cases, or when invasive respiratory support is needed soon after birth, clinicians must carefully choose a mode of mechanical ventilation.

Time-cycled pressure-limited ventilation (PLV) has been traditionally used for newborn infants. This form of ventilation uses a designated volume of gas with a preset peak inspiratory pressure (PIP), over a defined time cycle. Both overexpansion (volutrauma) and under expansion/collapse (atelectrauma) have been previously reported during the use of PLV (70–72). It has also been reported that tidal volume (V_T_), rather than inflation pressure, is the main determinant of ventilator-induced lung injury (VILI) (73). In volume-targeted ventilation (VTV), automatic adjustments are made to the peak positive pressure and the duration of the ventilator cycle to maintain a target V_T_. VTV mode has been proposed as a means to reduce VILI caused by ventilation with excessive or insufficient V_T_ during conventional pressure-controlled ventilation. A 2017 Cochrane review provided moderate-quality evidence supporting the use of volume-targeted ventilation over pressure-limited ventilation to reduce the composite outcome of death or BPD, length of mechanical ventilation, and rates of severe intraventricular hemorrhage (57).

High-frequency oscillatory ventilation (HFOV) is another ventilation strategy that may help mitigate lung injury. A 2015 Cochrane review evaluating HFOV as a primary mode of invasive respiratory support found a small reduction in the risk for death or BPD, as well as BPD alone, among infants treated with HFOV compared to pressure-limited conventional ventilation (58). However, pulmonary air leaks, such as pneumothorax or pulmonary interstitial emphysema, were more common in infants treated with HFOV (58).

#### Surfactant administration

Endogenous pulmonary surfactant plays a crucial role in reducing surface tension at the air/liquid interface within the alveoli, thereby enhancing lung deflation stability (74). In extremely preterm infants, surfactant deficiency is a central aspect of the pathophysiology of neonatal respiratory distress syndrome (RDS) (75). Several older RCTs demonstrated that administration of exogenous surfactant, compared with mechanical ventilation alone, reduces rates of death or the need for supplemental oxygen 28 days after birth (the conventional definition of BPD at that time) (76–78).

To optimize the potential advantages of early surfactant administration without the harmful effects of prolonged invasive mechanical ventilation, Victorin et al. introduced the technique known as intubation, surfactant administration during brief mechanical ventilation, followed by extubation (INSURE) (79). Initial RCTs suggested that INSURE reduces the need for supplemental oxygen at 28 days of age. However, meta-analyses incorporating more recent trials have indicated that compared to CPAP, INSURE does not decrease the risk of death or BPD (RR 0.88, 95% CI 0.76–1.02) (80, 81).

Several techniques have been developed to administer surfactant avoiding traditional endotracheal intubation (82). Among these strategies, surfactant instillation via a thin catheter, commonly known as less invasive surfactant administration (LISA) has been extensively studied. Four RCTs conducted in extremely preterm infants compared LISA with endotracheal tube administration of surfactant (three trials vs. INSURE, one trial vs. continued mechanical ventilation after surfactant therapy), while one trial compared LISA with CPAP therapy alone (83–86). A recent meta-analysis (59) showed that LISA vs. INSURE reduced the risk for BPD (RR 0.65, 95% CI 0.51–0.82) and the rate of mortality (RR 0.76, 95% CI 0.58–1.00).

#### Oxygen saturation targets

Exposure to supraphysiological oxygen has been associated with BPD, making the definition of optimal oxygen saturation targets a critical area of study. Infants born at less than 30 weeks gestation randomized to a high-saturation target (95%–98%) have a significantly higher risk of needing supplemental oxygen at 36 weeks compared to those randomized to a target of 92%–94% [odds ratio (OR) 1.40, 95% CI 1.15–1.70] (87). An individual patient meta-analysis of the five RCTs of the Neonatal Oxygen Prospective Meta-Analysis (NeOProM) Collaboration examined restricted (85%–89%) vs. liberal (91%–95%) oxygen saturation targets in infants less than 28 weeks gestation. The analysis found no significant difference in the composite outcome of death or major neurodevelopmental outcomes, or severe visual problems at 18–24 months between the two groups (RR 1.04, 95% CI 0.98–1.09) (88). Significantly fewer infants in the restricted oxygen saturation target group received supplemental oxygen at 36 weeks PMA (RR 0.81, 95% CI 0.74–0.90), but there was also an increase in the risk of death (RR 1.17, 95% CI 1.04–1.31) and necrotizing enterocolitis (NEC) (RR 1.33, 95% CI 1.10–1.61) in this group (89). While further studies are needed to make definitive conclusions, some authors suggest maintaining oxygen saturation targets between 88% and 92%, with a higher alarm limit of 96% (90).

### Pharmacologic therapies

Despite the physiological and observational evidence linking invasive mechanical ventilation to the development of BPD, the beneficial effects of the respiratory support strategies described above are modest. Longitudinal data also suggest that the increased use of noninvasive respiratory support over time has not been accompanied by substantial improvements in BPD rates among surviving extremely preterm infants (91). Given the limited benefit of gentle ventilation techniques, pharmacologic therapies are becoming an essential component in ongoing efforts to reduce BPD rates (92). Drug therapies demonstrated in randomized controlled trials to reduce BPD are summarized below and in Table 3.

**Table 3:** Summary of randomized, controlled trial data on the effects of various medications for preventing death and/or bronchopulmonary dysplasia.

Medication	Outcome	Trials/N	Outcome rates		Relative risk (95% CI)
			Intervention	Control	
Non-corticosteroids					
Azithromycin (93)	BPD among survivors	3/310	50%	60%	0.83 (0.71–0.97)
Caffeine (94)	BPD among survivors	1/1,917	36%	47%	0.78 (0.70–0.86)
Vitamin A (IM) (95)	BPD among survivors	4/886	43%	50%	0.85 (0.74–0.98)
Corticosteroids					
Dexamethasone (<7 days of life) (96)	BPD among survivors	17/2,791	26%	36%	0.72 (0.63–0.82)
	Death or BPD	17/2,791	42%	48%	0.88 (0.81–0.95)
Dexamethasone (≥7 days of life) (97)	BPD among survivors	12/553	50%	66%	0.76 (0.66–0.87)
	Death or BPD	12/553	59%	78%	0.75 (0.67–0.84)
Hydrocortisone (<7 days of life) (96)	BPD among survivors	9/1,376	35%	38%	0.92 (0.81–1.06)
	Death or BPD	9/1,376	51%	56%	0.90 (0.82–0.99)
Budesonide (inhaled) (98)	BPD among survivors	1/369	28%	38%	0.74 (0.60–0.91)
Budesonide + Surfactant (intratracheal) (99)	BPD	2/381	25%	44%	0.57 (0.43–0.76)
	Death or BPD	2/381	39%	65%	0.60 (0.49–0.74)

BPD, bronchopulmonary dysplasia; CI, confidence interval; IM, intramuscular; N, total number of infants evaluated for the outcome.

#### Noncorticosteroid agents

##### Azithromycin

Azithromycin is a macrolide antibiotic that possesses both antimicrobial and anti-inflammatory properties (100, 101), making it a potentially attractive option for preventing BPD. In very preterm infants, *Ureaplasma* infection is linked to BPD development (102–104). Additionally, lung and systemic inflammation contribute to BPD pathophysiology (105, 106). Three small trials assessed azithromycin's efficacy in preventing BPD (93). A meta-analysis of these studies revealed a reduction in the risk for BPD and BPD/death among infants treated with azithromycin, irrespective of known *Ureaplasma* colonization or infection. However, the quality of evidence was low (93, 107). Furthermore, trials evaluating other macrolides have not shown benefits for preventing BPD (107, 108). Larger trials are necessary to establish the safety and efficacy of prophylactic azithromycin before recommending this therapy.

##### Caffeine

Caffeine is a central nervous system (CNS) stimulant of the methylxanthine class that primarily acts by inhibiting the adenosine receptors A1 and A2A in the brain (109, 110). In addition, caffeine improves diaphragmatic contractility and prevents diaphragmatic fatigue by increasing intracellular Ca^2+^ and responsiveness of the central and peripheral chemoreceptors to CO_2_, resulting in increased minute ventilation (111).

The Caffeine for Apnea of Prematurity (CAP) trial was a pivotal study in the field of neonatology (94). This multicenter RCT investigated the long-term impacts of caffeine administration, initiated before ten days of age (median: 3 days), on infants <1,500 g. The trial examined a combined outcome of death and neurodevelopmental progress at 18–21 months of corrected age. The findings confirmed the safety of prolonged caffeine use in premature infants and demonstrated a significant reduction in the incidence of BPD with early caffeine administration (*p* < 0.001). By preventing apnea of prematurity, caffeine reduces the need for intubation and promotes early, successful extubation of ventilated infants, thereby reducing mechanical ventilation-associated lung injury, a critical factor in the development of BPD. Additionally, the CAP trial revealed that only 33.8% of infants treated with caffeine required medical or surgical closure of patent ductus arteriosus (PDA), compared to 50.7% in the placebo group (*p* < 0.001). Follow-up data collected through age 11 years indicated sustained, long-term improvement in motor function with caffeine therapy (112).

The benefits of caffeine in BPD prevention also depend on the timing of initiation of therapy. Since the CAP trial, the definition of early caffeine administration has evolved and is now defined as caffeine administration before three days of age. A retrospective study found that initiating caffeine treatment within the first three days of life was significantly associated with lower rates of BPD and the combined outcome of death or BPD compared to late caffeine administration (≥3 days) (113). Several RCTs and meta-analyses have confirmed this finding (114–117). The National Institute for Health and Care Excellence (NICE) recommends routine caffeine use for all premature infants ≤30 weeks’ GA soon after birth (118).

##### Vitamin A

Vitamin A plays a crucial role in the growth and maturation of epithelial cells lining the respiratory tract (119, 120). Previous studies have indicated that preterm infants who develop BPD tend to have lower plasma vitamin A levels (121–123). Subsequent large-scale trials, such as a multicenter trial published in 1999, found that intramuscular injections of vitamin A during the first 4 weeks of age reduced rates of death or BPD and BPD alone among surviving extremely low-birthweight infants (124). Meta-analyses of these trial data have confirmed a small benefit for reducing BPD among survivors (95). However, recent observational studies have raised questions about the effectiveness of vitamin A in the current era, with some studies showing similar rates of BPD among infants who received vitamin A and untreated controls (125, 126). Ongoing research, including randomized controlled trials investigating enteral vitamin A, may help resolve these conflicting findings. However, until more definitive results are available, intramuscular vitamin A administration, if commercially available, remains a recommended evidence-based strategy to prevent BPD in extremely preterm infants.

#### Corticosteroids

The potent anti-inflammatory properties of corticosteroids make them a logical therapeutic agent for BPD prevention.

##### Dexamethasone (systemic)

Dexamethasone has been extensively studied for preventing BPD, with trials categorizing its use into early initiation within the first 8 days of age and late initiation thereafter. A recent Cochrane review highlighted that early dexamethasone therapy reduces BPD risk but elevates the risks for gastrointestinal perforation, hypertrophic cardiomyopathy, cerebral palsy (CP), and major neurosensory disability (96). Due to these adverse effects, early dexamethasone for BPD prevention is not recommended.

The risks and benefits of “late” dexamethasone are not as well-defined. Meta-analysis of available trial data indicates that initiating dexamethasone after the first week of age reduces the risk of BPD but is associated with short-term side effects like hyperglycemia, glycosuria, and hypertension (96). Unlike early use, recent meta-analyses have not shown clear evidence of increased risk of CP among surviving infants treated with late dexamethasone (96). However, long-term outcome evaluations in follow-up studies lacked adequate power, and the high rates of open-label dexamethasone use in these studies may obscure actual treatment effects (97, 127).

If a clinician opts to administer dexamethasone, they must then determine the dose and treatment duration. While there is a general consensus favoring the use of low, tapering doses administered for short periods (typically 1–2 weeks at most), there is limited robust data to guide these specific choices (128). One dosing regimen used in the discontinued Dexamethasone: A Randomized Trial (DART) study involved administering 0.89 mg/kg over 10 days (129). In this trial involving 70 very preterm infants receiving invasive mechanical ventilation, dexamethasone significantly improved rates of successful extubation (60% in the dexamethasone group vs. 12% in the placebo group) without evidence of long-term harm (129, 130). However, the risk for BPD was not significantly reduced in the dexamethasone-treated infants (odds ratio 0.58, 95% CI 0.13–2.66) (129).

##### Hydrocortisone (systemic)

Randomized trials of hydrocortisone involving human infants are limited. The PREMILOC trial, the largest among them, compared a 10-day course of low-dose hydrocortisone initiated within the first 24 h after birth with placebo in infants born at less than 28 weeks’ gestation (131). Hydrocortisone significantly improved BPD-free survival at 36 weeks’ PMA (60% vs. 51%; *p* = 0.04) (131). However, a subgroup analysis revealed a nearly twofold increase in the risk of late-onset sepsis among infants born at 24–25 weeks’ gestation treated with early hydrocortisone (131). Furthermore, hydrocortisone did not improve neurodevelopmental outcomes at 2 years despite reducing the incidence of death or BPD (132). Meta-analysis of all available trials initiating hydrocortisone in the first week of age indicated a reduction in the composite outcome of death or BPD with hydrocortisone therapy but no benefit for BPD among survivors (96). Gastrointestinal perforation was more common in the hydrocortisone-treated infants (96).

The STOP-BPD trial was a double-blinded RCT in which 372 infants <30 weeks and/or 1,250 g birthweight were randomly assigned to receive systemic hydrocortisone (72.5 mg/kg over 22 days) or a placebo. Infants were enrolled if they were dependent on mechanical ventilation (MV) in the 2nd week of life. There was no significant difference in the BPD incidence at 36 weeks’ PMA or the composite outcome of death or BPD at 36 weeks’ PMA with hydrocortisone use. However, death at 36 weeks’ PMA was higher in the placebo group. Despite an initial decrease in extubation failure with hydrocortisone, this difference was not seen 21 days after starting the treatment (133).

##### Budesonide (inhaled)

Inhaled corticosteroids offer the potential benefit of reducing inflammation in the lung without the adverse effects associated with systemic corticosteroids administration. Research has investigated the efficacy of four different inhaled steroids (budesonide, beclomethasone, fluticasone, and flunisolide) in preventing BPD through RCTs (134, 135). A meta-analysis of all trial data, incorporating all four steroids, revealed a reduced risk of BPD among surviving infants as well as the composite outcome of death or BPD in infants treated with inhaled corticosteroids (134). However, these favorable results are primarily driven by the NEUROSIS trial, which found that inhaled budesonide decreased BPD rates among survivors (98, 136). It's important to note that this benefit was accompanied by higher mortality among infants treated with budesonide, with similar rates of neurodevelopmental impairment observed between the two study groups (136). Although the cause of the increased mortality in the budesonide group remains unidentified, this concerning finding outweighs the observed benefit for BPD.

Two RCTs evaluated the usefulness of intratracheal budesonide combined with surfactant relative to surfactant therapy alone among very low birth weight infants with severe RDS (99). The combined therapy reduced the risk of death or BPD. Follow-up performed up to 3 years of age found no difference in motor or cognitive function between the groups (99). This promising finding awaits confirmation in larger trials before widespread use is recommended.

### Nutritional strategies

The ability to maintain lung growth and repair is dependent on adequate postnatal nutrition. A retrospective cohort study revealed that both lower energy intake during the first four weeks of life and increased fluid intake were significantly associated with BPD (137).

Breast milk is well known for its protective effect against NEC, and has also been studied for its role in preventing BPD (138–140). In a multicenter cohort study of 1,587 preterm infants who received an exclusively human breast milk-based diet, the incidence of BPD was significantly lower compared to infants who received either preterm formula or maternal breast milk with bovine fortifier (56.3% vs. 47.7%, *p* = 0.0015) (140). A pooled meta-analysis of eight observational studies showed a BPD protective effect of donor human milk compared to formula when used as a supplement to the mother's own milk (RR 0.78, 95% CI 0.67–0.90) (140). However, a meta-analysis of three RCTs found no statistically significant difference in BPD between infants receiving donor human milk compared to preterm formula when the mother's own milk was unavailable (RR 0.89, 95% CI 0.60–1.32) (139).

### Unproven interventions

Certainly, while many medications and care strategies hold promise for preventing BPD, several have ultimately been found ineffective in reducing its risk through RCTs. Although delving into each of these therapies is beyond the scope of this article, it's worth mentioning a few of the more commonly considered strategies.

#### Antenatal corticosteroids

In 1972, Liggins et al. demonstrated that antenatal corticosteroids (ACS) promote lung maturation and prevent RDS in preterm infants (141). Approximately two decades later, the National Institute of Health (NIH) consensus panel recommended the use of ACS for all impending preterm births between 24 and 34 weeks of gestation (142). ACS promote lung maturation through several mechanisms. They induce the differentiation of alveolar epithelial cells into type II pneumocytes and increase the expression of surfactant proteins (SP)-A and SP-B, which enhances overall surfactant production. Additionally, ACS improve pulmonary blood flow by activating endothelial nitric oxide synthase and increase the activity of epithelial sodium channels, thereby improving respiratory function (143–146).

The American College of Obstetrics and Gynecology (ACOG) recommends specific regimens for ACS administration. For pregnant women between 24 0/7 and 33 6/7 weeks of gestation at risk of delivery within seven days, the recommended regimen is either two doses of intramuscular (IM) betamethasone or four doses of IM dexamethasone (147). In cases of pregnancies between 23 0/7 and 23 6/7 weeks of gestation, ACS should be administered if resuscitation is desired. Additionally, for women between 34 0/7 and 36 6/7 weeks of gestation who are at risk of preterm delivery within seven days and have not previously received ACS, administration is also recommended (147). Despite a clear improvement in mortality and RDS, the 2020 Cochrane review comparing ACS with placebo noted no significant difference in the incidence of BPD between the two groups (148).

#### Treatment of a patent ductus arteriosus

Observational data strongly suggest a link between the presence of a PDA and the development of BPD in preterm infants (149, 150). However, despite this association, no medication specifically targeting ductal closure (indomethacin, ibuprofen, or acetaminophen) administered either prophylactically or after identifying a “hemodynamically significant” PDA, has been shown to reduce the risk of BPD (151–155). Surgical ligation is effective in achieving closure of the PDA but may increase the risk for BPD and long-term neurodevelopmental impairment (156, 157). Given the lack of conclusive evidence surrounding PDA closure to prevent BPD and the adverse effects of all available management strategies, there is an increasing trend towards a more conservative approach of “watchful waiting” across centers (158).

#### Fluid restriction and diuretics

Excessive fluid intake in extremely low-birthweight infants can lead to complications such as pulmonary edema, necessitating greater respiratory support and potentially contributing to the development of BPD. Observational data have suggested that infants receiving higher fluid intake, particularly those with less weight loss in the first 1–2 weeks of life, are at an increased risk of developing BPD (150). However, studies comparing restrictive fluid administration with more liberal approaches have not consistently shown clear benefits in reducing the incidence of BPD (159). While restrictive fluid strategies may help mitigate the risk of pulmonary edema, their impact on BPD risk remains uncertain.

Diuretics are sometimes used to manage pulmonary edema in preterm infants, offering short-term improvement in respiratory mechanics. However, there is a lack of data indicating that regular diuretic use reduces the risk of developing BPD (160).

#### Inhaled nitric oxide

Inhaled nitric oxide (iNO) is a potent pulmonary vasodilator and an effective treatment for persistent pulmonary hypertension in near-term and full-term newborns (161). Despite these benefits, iNO does not prevent BPD when used as an early routine strategy or as a rescue therapy in very preterm infants (162, 163). A Cochrane review examining the efficacy of iNO in preterm infants included seventeen RCTs (163). The review found no significant difference in the overall incidence of death and/or BPD at 36 weeks’ PMA between preterm infants who received routine iNO and those in the control group (163–167). Furthermore, the recently published NEWNO trial, a large RCT involving 451 preterm infants less than 30 weeks’ GA and less than 1,250 grams birth weight, receiving mechanical ventilation between postnatal days 5 and 14, showed no significant differences in BPD at 36 weeks’ PMA or in neurodevelopmental and respiratory outcomes at 18–24 months between the treatment and placebo groups (168). Data from studies published to date are not sufficient to recommend the routine use of iNO for the prevention of BPD.

#### Bronchodilators

Data on the use of bronchodilators are limited. In a systematic review, only one randomized trial had usable outcome data. In this trial of 173 preterm infants (gestational age less than 31 weeks), salbutamol did not reduce the risk of BPD at 28 days when compared to no intervention/placebo (RR 1.03, 95% CI 0.78–1.37) (169).

### Experimental drugs

#### Stem cell therapy

Mesenchymal stromal cell (MSC) therapy is a relatively new treatment that may prove to be an essential weapon in the prevention of BPD. Multiple preclinical studies have demonstrated the promising reparative potential of stem/progenitor cells in promoting lung growth and preventing lung injury (170). In animal models, various types of progenitor cells have shown protective effects against neonatal lung injuries induced by factors such as lipopolysaccharide and hyperoxia (171). Importantly, these cell-based therapies primarily exert their effects through paracrine mechanisms rather than direct integration into the repaired tissue (172). Stem cells can modulate innate and adaptive immune responses, reduce inflammation, enhance injury repair, and exert anti-apoptotic effects by secreting paracrine factors (173).

A phase I dose-escalation study conducted in 2014 investigated the safety and feasibility of a single intratracheal transplantation of allogeneic human umbilical cord blood (hUCB)-derived mesenchymal stem cells in extremely low birth weight infants (ELBWI) at high risk for BPD (174). The study reported a decrease in the severity of BPD in the transplanted group compared to the control group, with no adverse outcomes observed. While this study suggested that hUCB-MSC therapy is safe and feasible in preterm infants, further research is necessary to fully elucidate the safety profile and efficacy of stem cell therapy or their conditioned medium before they can be utilized in clinical settings.

#### Erythropoietin (EPO)

Recombinant human erythropoietin (rhEPO) treatment has shown promise in neonatal rats exposed to hyperoxia, where it upregulated epidermal growth factor-line domain 7 and was associated with decreased alveolar simplification, improved angiogenesis, and decreased fibrosis (175, 176). However, in preterm lambs, EPO administration increased alveolar inflammation and exacerbated ventilator-associated lung injury (177). In a multi-center prospective cohort study involving 867 neonates born before 28 weeks of GA, those with higher blood EPO concentrations on day 14 of life had a higher incidence of moderate but not severe BPD (178).

While retrospective studies have suggested a potential benefit of erythropoietin in reducing BPD in preterm infants, a meta-analysis of 17 RCTs found no difference in the incidence of BPD between infants receiving EPO and those receiving placebo (179–181). The PENUT trial (Preterm Erythropoietin Neuroprotection trial) also showed no reduction in the incidence of BPD after high-dose EPO supplementation (182). Importantly, EPO use did not increase the incidence of retinopathy of prematurity (ROP), sepsis, or NEC (181, 182). Combining EPO with mesenchymal stem cells has shown potential in attenuating lung injury by promoting angiogenesis and decreasing fibrosis in murine hyperoxia-induced lung injury models (183, 184). However, this combination therapy has not yet been studied in humans.

#### Recombinant human Clara cell 10 protein (rhCC10)

Clara Cell 10 Protein (CC10) is a 10-kilodalton protein secreted by non-ciliated bronchiolar epithelial cells (club cells) and is one of the most abundant proteins within the fluid lining the lung epithelium (185). CC10 has extensive anti-inflammatory properties and has been shown to be significantly lower in tracheal aspirates of premature infants who subsequently died or developed BPD (186, 187). Animal studies have demonstrated that the administration of recombinant human CC10 (rhCC10) upregulates SP and vascular endothelial growth factor (VEGF) expression while improving respiratory mechanics (188, 189). A pilot trial conducted in 22 VLBW infants demonstrated that intratracheal administration of rhCC10 was well-tolerated and had significant anti-inflammatory effects in the lung (190). In another multicenter RCT consisting of 88 infants 24 0/7 to 29 0/7 weeks’ GA, a single dose of intratracheal rhCC10 (1.5 mg/kg or 5 mg/kg) was found to be ineffective in changing long-term respiratory outcomes at 12 months of age (191).

#### Superoxyde dismutase (SOD)

Superoxide dismutases (SODs) are antioxidant enzymes that are reduced in various animal models of BPD (192, 193). Studies have shown that increased expression of extracellular SOD in transgenic mice preserved pulmonary angiogenesis following exposure to hyperoxia (194). Initial findings by Rosenfeld et al. suggested a decrease in both radiologic and clinical features of BPD after the subcutaneous administration of multiple doses of bovine SOD to infants with severe RDS (195). However, a randomized controlled trial by Davis et al. did not find a difference in the rate of death or BPD at 28 days or 36 weeks of PMA in infants receiving recombinant human CuZn-SOD (rh-CuZn-SOD) compared to placebo. Notably, infants treated with rhCuZn-SOD experienced fewer episodes of wheezing, required asthma medications less frequently, and had lower hospitalization rates before one year of corrected age (196). A recent study found no correlation between extracellular SOD in serum and the risk of BPD in infants born at less than 32 weeks’ gestational age (197). While SODs may hold promise for improving the long-term pulmonary outcomes of infants with BPD, further research is needed to determine their efficacy, safety, and optimal dosing before they can be implemented in clinical practice.

#### Inositol

Inositol is a nutrient that promotes the production of phosphatidylcholine and phosphatidylinositol, and preterm infants with a premature decline in myoinositol have a more severe course of RDS (198). Multiple RCTs have been conducted to evaluate the efficacy of inositol supplementation in preventing RDS and BPD. While no acute adverse effects were reported with the use of inositol, a recent Cochrane review concluded that inositol supplementation did not decrease the rates of BPD, death, or the composite outcome of BPD or death in preterm infants. Based on these findings, the review recommended against further clinical trials in neonates regarding the use of inositol for this purpose (199, 200).

#### Docosahexaenoic acid (DHA)

DHA is a long-chain polyunsaturated fatty acid known for its anti-inflammatory and antioxidant properties (201). In the DINO trial, which involved over 600 infants born before 33 weeks of GA, Manley et al. observed a reduced incidence of BPD in male infants and those weighing less than 1.25 kg whose mothers consumed tuna oil capsules containing a high amount of DHA. However, no significant difference was observed in female infants or those weighing more than 1.25 kg (202). However, the N3RO trial, which included over a thousand infants born before 29 weeks’ GA, reported a higher rate of BPD at 36 weeks of PMA and of BPD or death before 36 weeks’ PMA in infants receiving enteral DHA supplementation at a dose of 60 mg/kg/day (203). Similarly, the MOBYDIck trial, which involved mothers delivering infants at less than 29 weeks’ GA, found no significant improvement in BPD-free survival rates in infants whose mothers received DHA capsules (204). Due to insufficient data and conflicting results, DHA should only be used in research settings. Larger clinical studies are necessary to determine the appropriate dose and assess the efficacy of DHA in preventing BPD in extremely preterm infants.

#### Other potential treatments

Various potential targets including growth factors, anti-inflammatory agents, and antioxidants are currently being evaluated for the prevention of BPD in extremely premature infants. In a phase II RCT that included infants with a gestational age of 23 weeks and 0 days to 27 weeks and 6 days, Ley et al. demonstrated a significant decrease in the incidence of severe BPD following treatment with recombinant human insulin-like growth factor 1 (rhIGF-1) complexed with recombinant human insulin-like growth factor-binding protein 3 (rhIGFBP3) (205). Additionally, other factors showing promise in the pre-clinical phase include growth factors such as hypoxia-inducible factor 1-alpha (HIF-1α) and vascular endothelial growth factor (VEGF), anti-inflammatory agents like interleukin-1 receptor antagonist (ILR1A), and various long non-coding and micro RNAs (lncRNA and miRNA) (206–210).

## Conclusions

BPD requires comprehensive strategies for prevention throughout the neonatal period. Initial respiratory management of very preterm infants should prioritize noninvasive support like nasal CPAP, reserving endotracheal intubation and surfactant administration for those who fail noninvasive support or do not demonstrate spontaneous respiratory effort after resuscitation. Utilizing a volume-targeted ventilation approach for those requiring mechanical ventilation may reduce the risk of BPD. Caffeine and vitamin A are supported by strong evidence for BPD prevention. Dexamethasone may be considered in high-risk cases initiated after the first week, although its risks should be carefully weighed. Hydrocortisone is an alternative with benefits but potential adverse effects. Newer experimental therapies appear to be promising for BPD prevention in extremely preterm infants; however, further research is necessary before their safety and efficacy in clinical practice can be established.
